# Outcomes of Direct Vision Internal Urethrotomy for Bulbar Urethral Strictures: Technique Modification with High Dose Triamcinolone Injection

**DOI:** 10.1155/2015/281969

**Published:** 2015-10-21

**Authors:** Rishi Modh, Peter Y. Cai, Alyssa Sheffield, Lawrence L. Yeung

**Affiliations:** Department of Urology, University of Florida College of Medicine, Gainesville, FL 32608, USA

## Abstract

*Objective*. To evaluate the recurrence rate of bulbar urethral strictures managed with cold knife direct vision internal urethrotomy and high dose corticosteroid injection. *Methods*. 28 patients with bulbar urethral strictures underwent direct vision internal urethrotomy with high dose triamcinolone injection into the periurethral tissue and were followed up for recurrence. *Results*. Our cohort had a mean age of 60 years and average stricture length of 1.85 cm, and 71% underwent multiple previous urethral stricture procedures with an average of 5.7 procedures each. Our technique modification of high dose corticosteroid injection had a recurrence rate of 29% at a mean follow-up of 20 months with a low rate of urinary tract infections. In patients who failed treatment, mean time to stricture recurrence was 7 months. Patients who were successfully treated had significantly better International Prostate Symptom Scores at 6, 9, and 12 months. There was no significant difference in maximum flow velocity on Uroflowmetry at last follow-up but there was significant difference in length of follow-up (*p* = 0.02). *Conclusions*. High dose corticosteroid injection at the time of direct vision internal urethrotomy is a safe and effective procedure to delay anatomical and symptomatic recurrence of bulbar urethral strictures, particularly in those who are poor candidates for urethroplasty.

## 1. Introduction

Epidemiological data suggests that patients with urethral strictures most commonly present with voiding complaints such as weak stream (49%) and incomplete emptying (27%) and the main causes are idiopathic, iatrogenic (endoscopic procedures, catheterization, prostatectomy, brachytherapy, and hypospadias repair), and traumatic [1, 2]. Management of urethral stricture ranges from commonly performed procedures such as urethral dilation and internal urethrotomy to more definitive reconstructive procedures such as urethroplasty or even urinary diversion in an effort to prevent complications from untreated strictures.

Currently, the most commonly employed procedures for treatment of urethral strictures are dilation and internal urethrotomy [2]. Studies have demonstrated that there is no significant difference between dilation and internal urethrotomy in complications or failure rates [3]. The decision to employ one technique over the other is often based on clinician preference and location of the stricture.

Although these procedures can be done relatively quickly in an ambulatory setting, one of the major concerns is the high rate of recurrence on long-term follow-up. Historical data shows recurrence rates for 2–4 cm strictures to be 50% within 12 months of urethrotomy [3]. Pansadoro and Emiliozzi demonstrated that, with long-term follow-up, a single urethrotomy had a recurrence rate of 68% [4]. The same group demonstrated that those with longer strictures (>1 cm), a narrow lumen (<15 French), or a history of prior interventions are more likely to recur after urethrotomy.

One proposed solution to decrease stricture recurrence rates is the use of adjunctive corticosteroid injections, which have been used to inhibit scar formation. Corticosteroid injections have been used in other specialties such as in Dermatology for hyperplastic and hypertrophic skin disorders, in Gastroenterology for esophageal strictures, and in Otolaryngology for laryngeal strictures.

Two studies have reported improved patency rates and delayed stricture recurrence with DVIU and steroid injection compared to DVIU alone [5, 6]. These studies utilized periurethral injection of triamcinolone at a dose of 40 mg after urethrotomy. We hypothesized that DVIU of bulbar urethral strictures with higher dose intralesional triamcinolone (320 mg) can significantly delay urethral stricture recurrence.

## 2. Materials and Methods

We reviewed our Institutional Review Board approved database of patients who underwent DVIU by a single surgeon (L. L. Yeung). All patients who underwent DVIU or dilation and corticosteroid injection for bulbar urethral strictures were included in our study. Patients who did not follow up for at least 6 months, those with penile urethral strictures, or those with multiple strictures were excluded. Every patient was evaluated preoperatively with cystoscopy and retrograde urethrogram to define length and location of stricture and subsequently offered urethroplasty, DVIU with steroid injection, or dilation with steroid injection based on location and length of stricture.

Our follow-up protocol was every 3 months for the first year, every 6 months for the second year, and then yearly with International Prostate Symptom Score (IPSS) and Uroflowmetry and postvoid residual. In order to establish an objective outcome measure, we defined successful treatment as maximum flow velocity greater than 15 mL/sec on Uroflowmetry. Patients who performed clean intermittent self-catheterization preoperatively for incomplete bladder emptying due to myogenic failure were considered a success if they were still able to perform self-catheterization without difficulty on follow-up. For those with equivocal Uroflowmetry rates, cystoscopy was performed to evaluate recurrence. DVIU failure was defined by the need for a subsequent urethral procedure (i.e., urethroplasty, dilation, and DVIU).

Urethrotomy was performed using a Sachse Urethrotome (Karl Storz, USA) by performing radial cuts through the stricture at the 12, 3, 6, and 9 o'clock positions. Urethral dilation was performed using Amplatz Dilators (Cook Medical, USA) to 26 F. A 23 G Williams Cystoscopic Injection Needle (Cook Medical, USA) was used to inject 1 mL (40 mg) triamcinolone at each site every 5–10 mm circumferentially in quadrants along the length of the incised stricture. The Encore 26 Inflator (Boston Scientific, USA) was used to provide positive pressure to aid in the injection of the steroids into the stricture and to provide accurate dose delivery. Although a total of 10 mL of triamcinolone (concentration of 200 mg/5 mL) was available for injection, 8 mL (320 mg) was injected after accounting for waste within the injection needle and tubing from the pressure inflator.

## 3. Results

In our cohort, we were able to identify twenty-eight patients with a single bulbar urethral stricture who had follow-up of at least 6 months after their initial procedure (Table 1(A)). Since our patients are from a tertiary care center, our cohort tended to be older with average of 60 years; 71% had prior procedures (urethrotomy, dilation, or urethroplasty) and on average had 5-6 prior procedures. Our cohort was at high risk for recurrence based on these characteristics. Patients with longer (>1 cm) or recurrent strictures included in our study either declined urethroplasty or were not surgical candidates for reconstructive procedures.

As seen in Table 1(B), the majority of the strictures were iatrogenic (32% radiation induced, 28% from prior endoscopic procedures, and 11% from traumatic catheterization). The remaining patients either experienced pelvic trauma or did not have an identifiable cause. Our cohort only experienced a recurrence rate of 29% with 20 months of follow-up (Table 1(C)). Failures tended to recur early with a mean of recurrence of 7 months. While 14% of patients were treated for a lower urinary tract infection (Table 1(D)), no other significant complications were noted.

**Figure 1:**
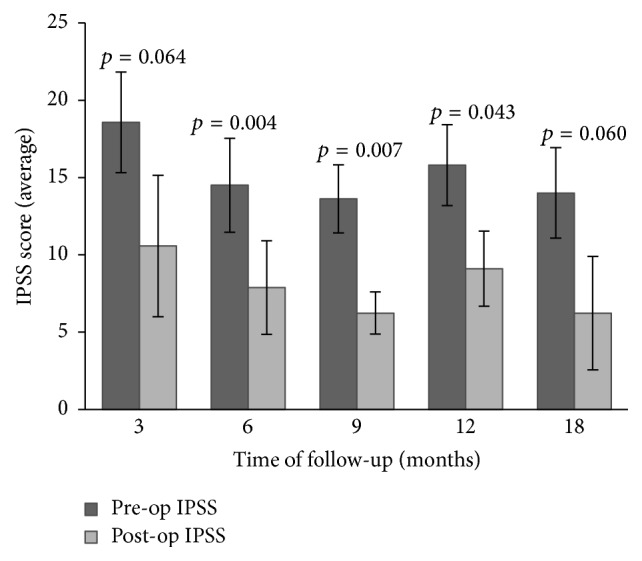
Preoperative versus postoperative IPSS at different follow-up times (error bars represent standard error of mean).

Our study suggests that patients who failed DVIU treatment had greater number of prior procedures and longer length of strictures, although statistical significance was not achieved in our sample size (Table 2). We were able to collect maximum flow velocity data on 19/20 patients who had successful DVIU treatment and 5/8 patients who failed DVIU treatment. Incomplete data was mainly due to loss of follow-up, presence of suprapubic tubes, and no urge for urination. Overall, no significant difference (*p* = 0.34) in the last recorded maximum flow velocity was found in patients who were successfully treated (14.90 mL/sec) versus those who failed treatment (13.06 mL/sec). However, there was a significant difference (*p* = 0.02) in follow-up time with those who were successfully treated seen for an average of 500.11 days after treatment while those who failed treatment were seen for an average of 143.80 days after treatment. In addition, analysis of IPSS represented in Figure 1 shows that there was a general trend of decreased IPSS score at all 5 time-points (3, 6, 9, 12, and 18 months) with statistical significance at 6, 9, and 12 months.

## 4. Discussion

The injection of steroids has also been used to decrease the recurrence of scarring in other fields of medicine. Studies on wound healing of oral mucosa suggest that there is more rapid and scarless healing in mucosal wounds compared to dermal wounds due to differences in expression of extracellular matrix components, immune mediators, and profibrotic mediators, as well as structural differences in blood vessels, mesenchymal stem cells, and fibroblast proliferation rate [7, 8]. Steroids are hypothesized to reduce scar formation by reducing the rate of collagen synthesis in fibroblasts during the wound healing process [9], which may be the mechanism by which steroid injection after urethrotomy delays urethral stricture recurrence. Different doses and methods of delivery have been used for steroids in the management of urethral strictures. Our technique modification uses higher doses of triamcinolone injection after DVIU and an accurate mechanism for delivery. High dose steroids (400 mg) have previously been used in the bladder for treatment of Hunner's ulcer subtype interstitial cystitis with no complications noted [10]. Further randomized studies are needed to determine the ideal dose of steroids.

DVIU is a simple and effective procedure for the management of short urethral strictures. However, stricture recurrence is a significant problem after DVIU particularly for longer and recurrent strictures. We evaluated the efficacy of DVIU with a higher dose of intralesional steroids for all patients who were not able or willing to undergo urethroplasty, regardless of stricture length, prior interventions, or etiology.

Previous studies on intralesional steroid injection after DVIU also demonstrated significantly improved success rates and delayed stricture recurrence compared to DVIU alone. One randomized control trial in fifty male patients showed decreased recurrence rates in the 40 mg triamcinolone group (21.7%) versus control group (50%) after a mean follow-up time of 13.7 ± 5.5 months [5]. Another double-blind, randomized, placebo-controlled study of 70 patients showed that the triamcinolone group versus control group had significantly decreased time to recurrence, 8.08 ± 5.55 months versus 3.6 ± 1.59 months with no evidence of complications from steroid injection [6]. The former study included only patients with bulbar urethral strictures and the later study included 61.42% bulbar and 28.57% penile strictures. Both of these studies only included patients with short strictures and no prior interventions. Another recently published systematic review showed that, in 203 patients across 8 studies, DVIU with corticosteroids were shown to have statistically significant decreased time to recurrence compared to DVIU alone (10.14 versus 5.07 months, *p* < 0.00001) [11].

At our institution, patients identified for our cohort were based on previous evidence endorsing the use of IPSS greater than 15 and maximum flow velocity less than 15 mL/sec as parameters to maximize sensitivity (91%) and specificity (72%) in order to detect the most men with strictures while also excluding a significant portion of those without disease to avoid further invasive testing [12]. Our cohort provides more evidence regarding the efficacy of intralesional steroid injection after DVIU by demonstrating a low recurrence rate (29%) with mean follow-up length of 20 months in patients with a mean of 5.7 prior procedures. In comparison to historical data on patients with no prior intervention, Steenkamp et al. (40% for strictures less than 2 cm, 50% for strictures 2 to 4 cm, and 75% for strictures greater than 4 cm at 12 months) and Pansadoro and Emiliozzi (68% overall and 89% for bulbar urethral strictures at median follow-up of 98 months) showed higher recurrence after single urethrotomy [3, 4]. We are able to demonstrate good efficacy with higher dose steroid injection, especially in patients with long and recurrent strictures. Of the 7 patients, or 25% of the total cohort, who did not have any prior stricture interventions, all of them were successfully treated at a mean follow-up length of 21 months (data not shown).

While our cohort had no significant difference (*p* = 0.34) in the last recorded maximum flow velocity in patients who were successfully treated (14.90 mL/sec) versus those who failed treatment (13.06 mL/sec), there was a significant difference (*p* = 0.02) in follow-up time (500.11 days for successfully treated versus 143.80 days for failed treatment). These results suggest that while final functional outcome may not be improved, successful treatment with DVIU and steroid injection can successfully delay recurrence. We included all Uroflowmetry data collected at clinic in order to minimize selection bias regardless of voiding volume. However, we recognize that a minimum voiding volume of at least 150 mL is often used to avoid inaccurate Uroflowmetry results [13]. In the patients with successful DVIU, patients with maximum flow velocity less than 15 mL/sec (*n* = 10) had an average voiding volume of 109 mL, whereas patients with maximum flow velocity more than 15 mL/sec (*n* = 9) had an average voiding volume of 392 mL (*p* = 0.00). This suggests that one reason why our cohort had no significant difference in maximum flow velocity may be due to low voiding volumes and emphasizes the importance of having patients be informed about needing to perform Uroflowmetry at clinic visits. When considering changes in IPSS scores, DVIU with steroids may help reduce symptoms secondary to urethral strictures at 6, 9, and 12 months but did not have as robust an effect in the subacute period (3 months) and beyond one year. These results altogether support our hypothesis that DVIU with steroids may help delay stricture recurrence and symptoms but does not serve as a permanent solution for urethral strictures.

Some limitations of our study include the retrospective nature of the study and the limited number of patients in our cohort. However, we were able to demonstrate similar efficacy of DVIU with steroid injection as seen in prior studies, despite this study having a more heterogeneous population with strictures at high risk for recurrence. In addition, as mentioned previously, strictures <1 cm have been associated with the highest rate of success [4]. Our study cohort included patients with longer strictures, even up to 4 cm in length, that are often not treated with DVIU. In general, these patients are not recommended to undergo DVIU due to low rates of success previously reported in the literature. However, we encountered patients who were not candidates for reconstructive surgery and after a patient-centered discussion with the appropriate counseling on the low rates of success for longer strictures, patients who were adamant about pursuing DVIU were granted that option. Another possible limitation is the use of noninvasive techniques (Uroflowmetry and postvoid residual) to monitor stricture recurrence. While performing cystoscopy and/or retrograde urethrography would be more definitive at detecting recurrences, these tests are invasive and patients are subjected to discomfort. Noninvasive methods of stricture surveillance with Uroflowmetry and postvoid residual are widely accepted amongst urologists [14].

## 5. Conclusions

In our series of patients treated with DVIU and high dose corticosteroid injections, we observed a recurrence rate of only 29% with an average follow-up of 20 month. DVIU with high dose steroids appears to be useful even in those who have had multiple prior interventions and those with longer bulbar urethral strictures. In addition, use of high dose corticosteroids was not associated with any significant adverse side effects. DVIU with high dose corticosteroid injections should be considered in the treatment algorithm of bulbar urethral strictures, particularly for men who are unwilling or unable to undergo urethroplasty. Future randomized control trials are needed to confirm these findings.

## Figures and Tables

**Table 1:** 

(A) Patient characteristics	
Number of patients	28
Mean age (range)	60 (24–90)
Mean American Society of Anesthesiologists (ASA) physical status classification	2.6
Mean number of prior procedures (range)	5.7 (0–50)
Percent with prior procedures	71%
Mean stricture length (range)	1.85 (0.5–4 cm)

(B) Stricture etiology	

Radiation	32% (9)
Endoscopic procedure	28% (8)
Pelvic trauma	18% (5)
Catheter trauma	11% (3)
Idiopathic	11% (3)

(C) Overall outcomes	

Number of patients	20
Stricture recurrence	8
Recurrence rate	29%

Patient on Uroflowmetry	14
Patient on cystoscopy	4
Patient on self-catheterization	2

Average follow-up	20 months
Time to recurrence for failures	7 months

(D) Complications	

Clavien Grade II: urinary tract infection	14%

**Table 2:** 

	Failure (*n* = 8)	Success (*n* = 20)
Comparison between treatment failures and successes		
Any prior procedure	100%	60%
Average number of prior procedures	9.8	4
	*p* = 0.18	
Average stricture length (cm)	2.2	1.7
	*p* = 0.16	

Etiology		
Radiation	50%	25%
Endoscopic	13%	35%
Pelvic trauma	25%	15%
Catheter trauma	13%	10%
Unknown	0%	15%

Uroflowmetry results		
Average maximum flow velocity (mL/sec)	13.06	14.90
	*p* = 0.34	
Average follow-up time (days)	143.80	500.11
	*p* = 0.02	
